# Supplementary figures and images for: Genome-Wide Characterization of Tomato FAD Gene Family and Expression Analysis under Abiotic Stresses
Source: Plants (Basel). 2023 Nov 10;12(22):3818. doi: 10.3390/plants12223818 (PMC10675527; doi:10.3390/plants12223818)

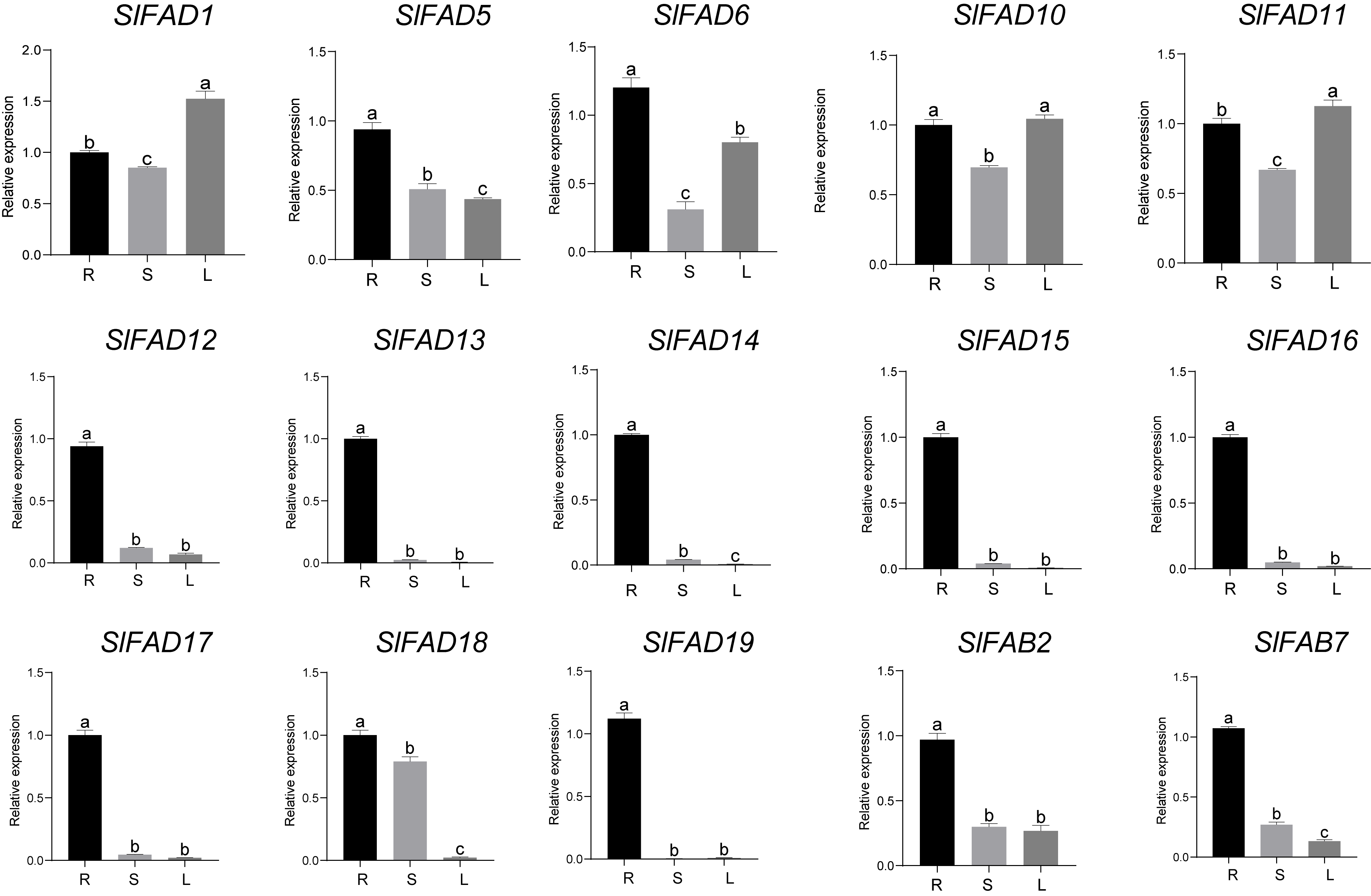

Supplement: Supplementary file 1 [file plants-12-03818-s001.zip › supplementary material/supplementary material/Figure S1.tif]

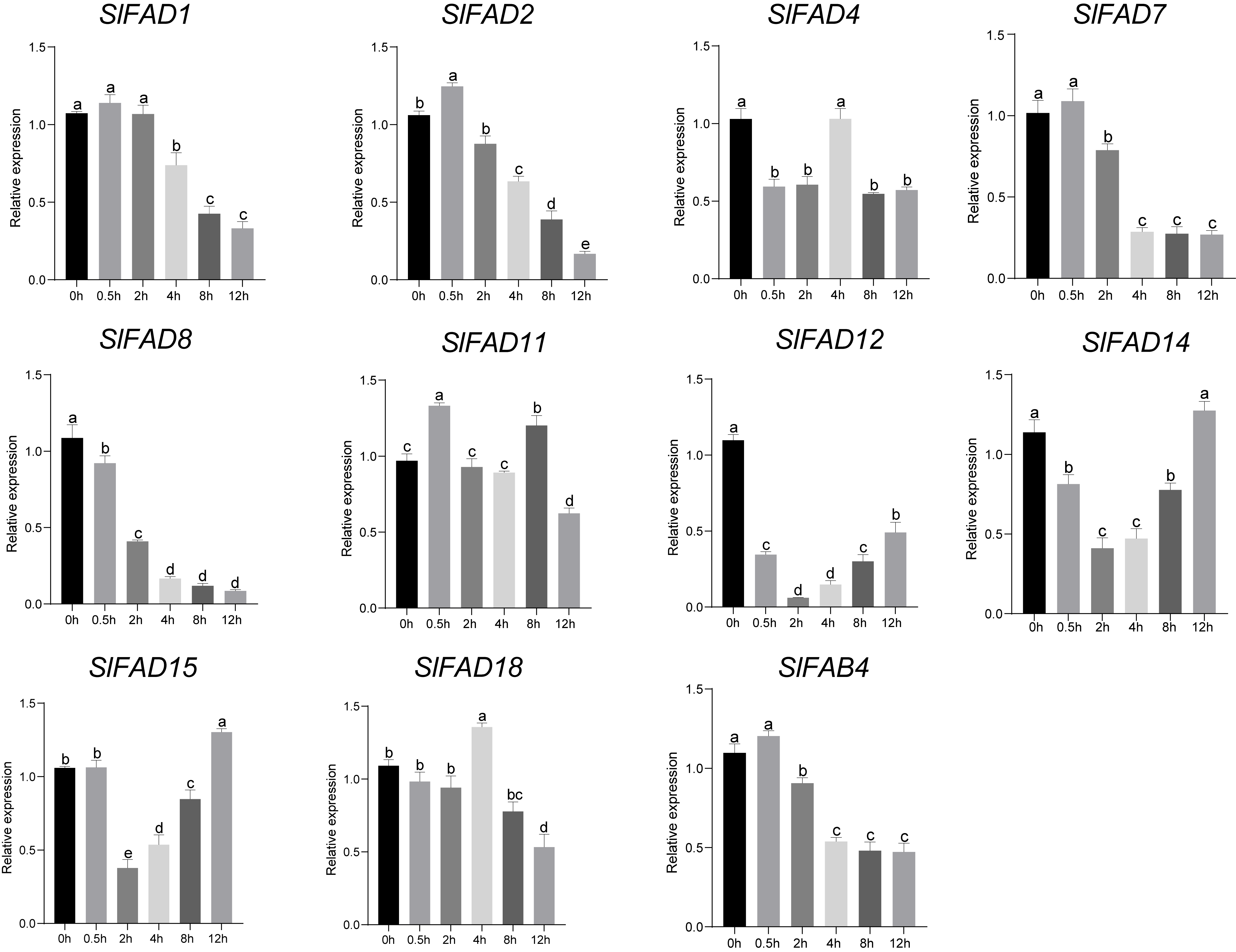

Supplement: Supplementary file 1 [file plants-12-03818-s001.zip › supplementary material/supplementary material/Figure S2.tif]

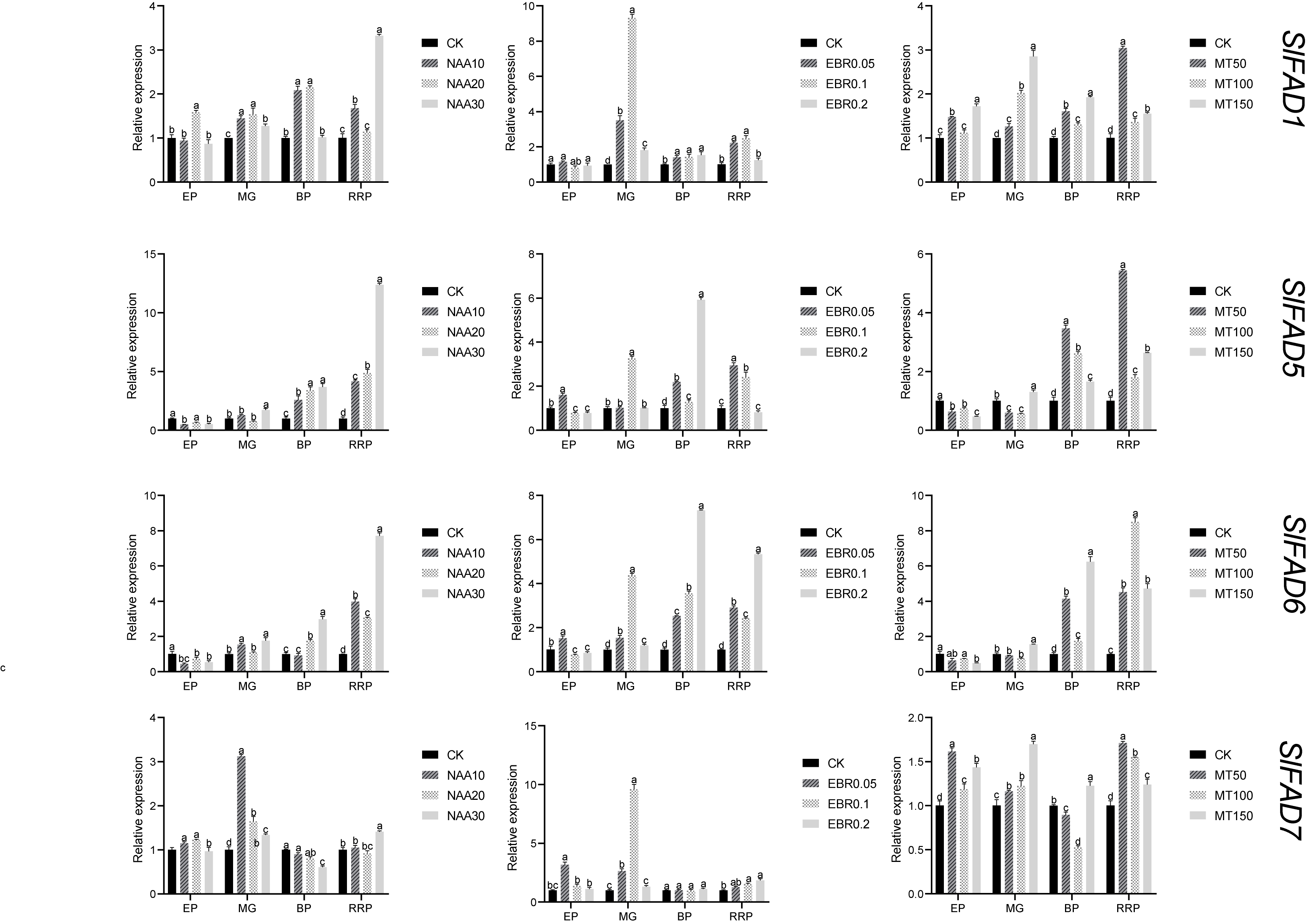

Supplement: Supplementary file 1 [file plants-12-03818-s001.zip › supplementary material/supplementary material/Figure S3.tif]

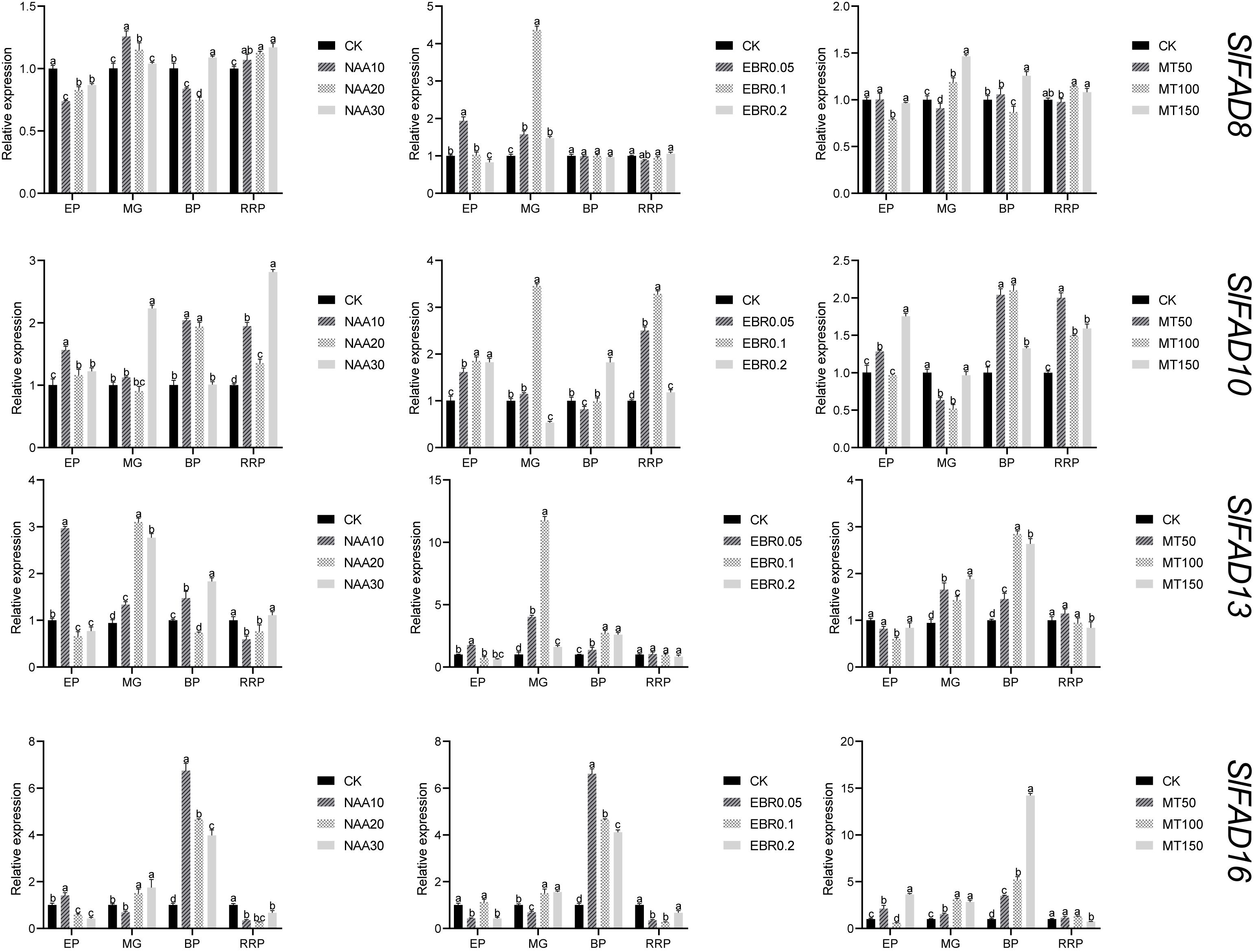

Supplement: Supplementary file 1 [file plants-12-03818-s001.zip › supplementary material/supplementary material/Figure S4.tif]

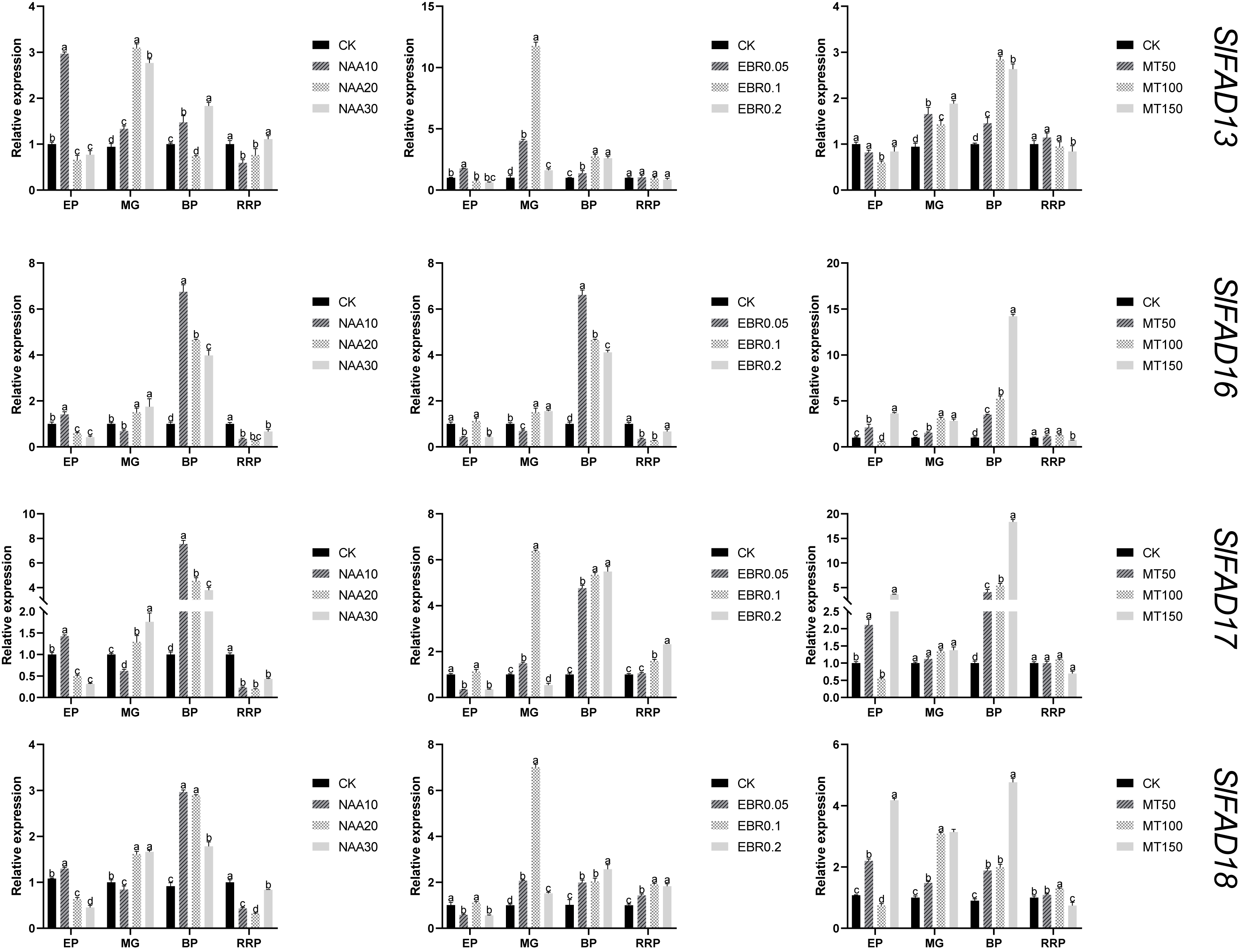

Supplement: Supplementary file 1 [file plants-12-03818-s001.zip › supplementary material/supplementary material/Figure S5.tif]

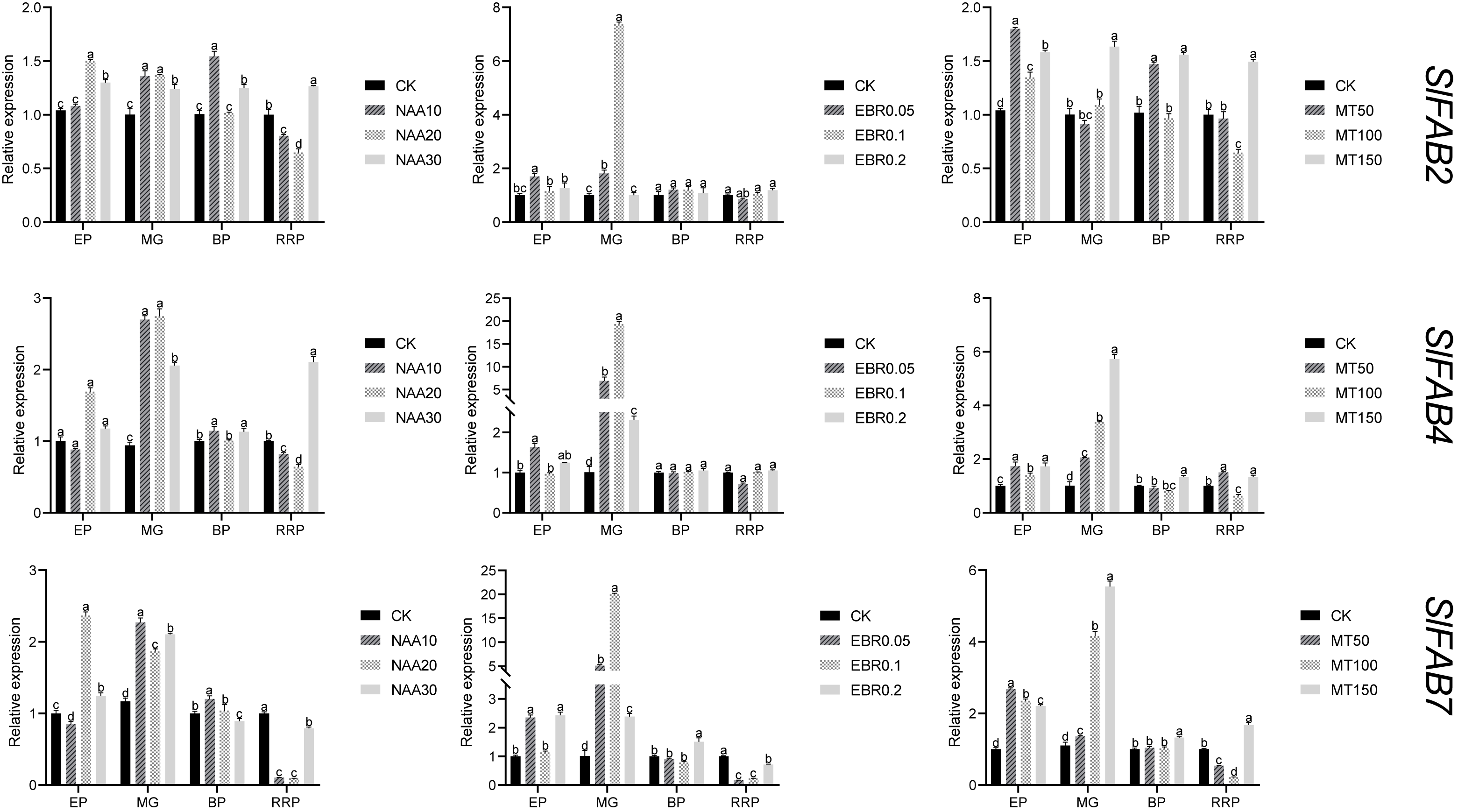

Supplement: Supplementary file 1 [file plants-12-03818-s001.zip › supplementary material/supplementary material/Figure S6.tif]
